# Benefits in radical mastectomy protocol: a randomized trial evaluating the use of regional anesthesia

**DOI:** 10.1038/s41598-018-26273-z

**Published:** 2018-05-18

**Authors:** Marcio Matsumoto, Eva M. Flores, Pedro P. Kimachi, Flavia V. Gouveia, Mayra A. Kuroki, Alfredo C. S. D. Barros, Marcelo M. C. Sampaio, Felipe E. M. Andrade, João Valverde, Eduardo F. Abrantes, Claudia M. Simões, Rosana L. Pagano, Raquel C. R. Martinez

**Affiliations:** 1 Hospital Sirio-Libanes, Rua Professor Daher Cutait, 69, 01308-060 Sao Paulo, Brazil; 2 Sao Paulo Servicos Medicos de Anestesia, Rua Adma Jafet, Jafet, 91 - Bela Vista, São Paulo - SP, 01308-050 Sao Paulo, Brazil

## Abstract

Surgery is the first-line treatment for early, localized, or operable breast cancer. Regional anesthesia during mastectomy may offer the prevention of postoperative pain. One potential protocol is the combination of serratus anterior plane block (SAM block) with pectoral nerve block I (PECS I), but the results and potential benefits are limited. Our study compared general anesthesia with or without SAM block + PECS I during radical mastectomy with axillary node dissection and breast reconstruction using evaluations of pain, opioid consumption, side effects and serum levels of interleukin (IL)-1beta, IL-6 and IL-10. This is a prospective, randomized controlled trial. Fifty patients were randomized to general anesthesia only or general anesthesia associated with SAM block + PECS I (25 per group). The association of SAM block + PECS I with general anesthesia reduced intraoperative fentanyl consumption, morphine use and visual analog pain scale scores in the post-anesthetic care unit (PACU) and at 24 h after surgery. In addition, the anesthetic protocol decreased side effects and sedation 24 h after surgery compared to patients who underwent general anesthesia only. IL-6 levels increased after the surgery compared to baseline levels in both groups, and no differences in IL-10 and IL-1 beta levels were observed. Our protocol improved the outcomes of mastectomy, which highlight the importance of improving mastectomy protocols and focusing on the benefits of regional anesthesia.

## Introduction

Breast cancer is the most common cancer in females, with 2.4 million incident cases worldwide^1^. The standard treatment options for early, localized, or operable breast cancer may include breast-conserving surgery and sentinel node biopsy with or without axillary lymph node dissection for positive sentinel lymph nodes or radical mastectomy (removal of the entire breast with axillary dissection of levels I and II) with or without breast reconstruction and sentinel node biopsy with or without axillary lymph node dissection for positive sentinel lymph nodes^2^.

One concerning problem that affects breast cancer patients after surgery is the pain. The incidence reaches 53% six months after the surgery, which emphasizes the importance of pain management^3^. One approach for pain management include the use of regional anesthesia^3^.

The use of regional anesthesia techniques may modulate the immune system, likely via interleukins (IL)^4^. Deegan reported increased IL-10 levels after propofol/paravertebral anesthesia for breast cancer compared to sevoflurane/opioids^5^. Additionally, IL-6 and IL-10 are important in the coordination of breast carcinogenesis^6,7^. The proinflammatory cytokines IL-1 beta and IL-6 are linked to breast cancer progression and have been hypothesized to be targets of anti-inflammatory drugs used to treat breast cancer^8,9^. IL-10 has prognostic value since its expression is related to recurrence, metastasis and poor survival in breast cancer^10–13^. As a therapeutic target, a high level of IL-10 has been associated with drug resistance of breast cancer^14^.

Blanco proposed alternative regional anesthesia techniques, including the serratus anterior plane block (SAM block) and pectoral nerve blocks I and II (PECS I and PECS II)^15–18^. The effectiveness of PECS for breast surgery was recently investigated and resulted in improved postoperative pain^19^. However, compared with PECS II, the SAM block is an easier and simpler technique that offers long-lasting regional anesthesia^18^. The combination of SAM block + PECS I may provide greater levels of analgesia for radical mastectomy with axillary lymph node dissection and breast reconstruction because of the pattern of analgesia, as previously suggested^20,21^; however, the results and potential benefits of SAM block + PECS I are very limited considering that these authors evaluated the effectiveness of this combination in only two patients, emphasizing the importance of further studies to validate this alternative surgical approach.

The evaluation of general anesthesia associated with SAM block + PECS I may provide important insights for an alternative anesthetic protocol for this type of mastectomy, but the results and potential benefits are limited. Thus, the aim of our study was to compare general anesthesia associated with SAM block + PECS I to general anesthesia only during radical mastectomy with axillary node dissection and breast reconstruction. The primary outcome measure was pain intensity measured before surgery, in the post-anesthetic care unit (PACU) and at 24 h after surgery. Additionally, it was evaluated opioid consumption, side effects and serum levels of IL-1beta, IL-6 and IL-10. Our results improved the outcomes of mastectomy, which highlight the benefits of regional anesthesia.

## Results

**Figure 1:**
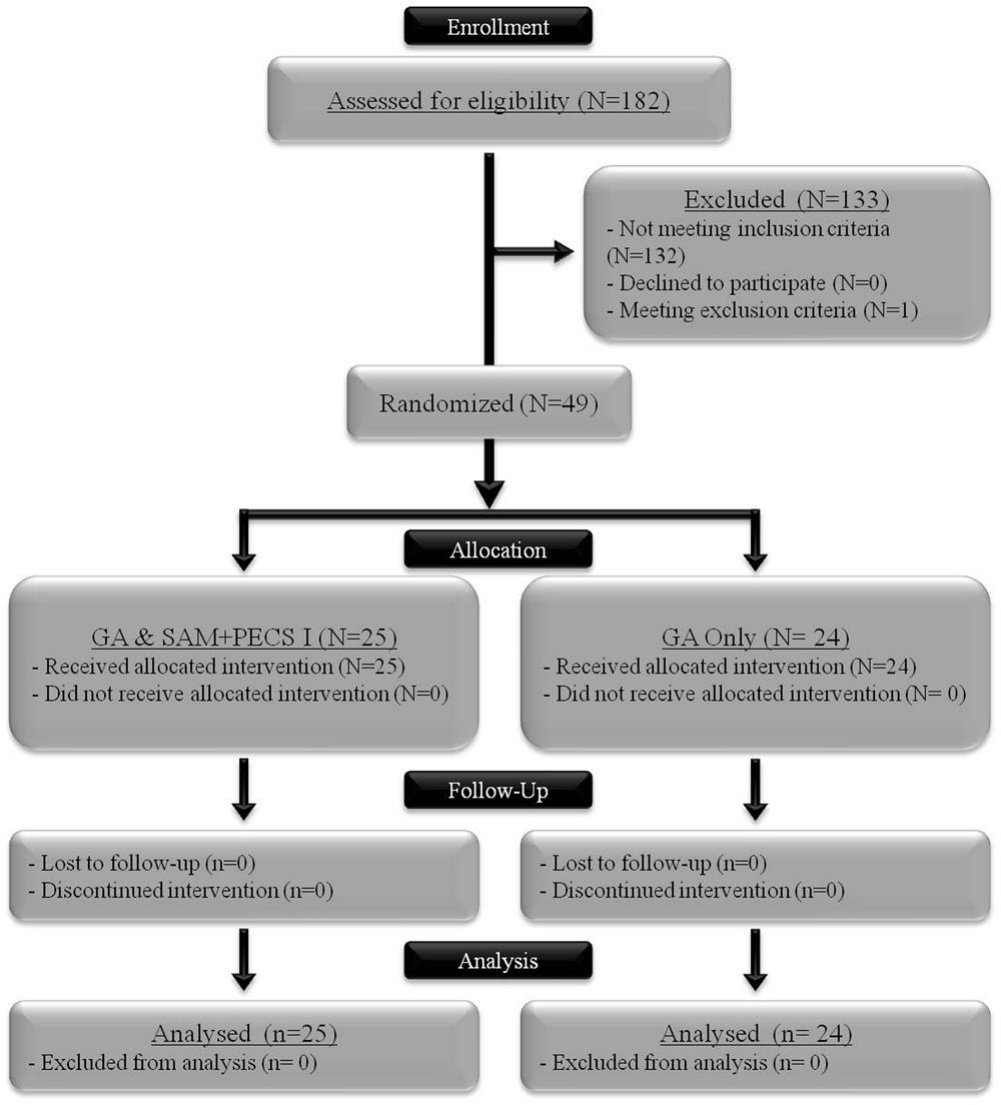
CONSORT flowchart of the surgeries performed during study development. GA: general anesthesia.

A total of 182 surgeries for breast cancer were performed from December 2015 to April 2016, and 133 cases were excluded because of different types of surgical procedures (i.e., mastectomy only, quadrantectomy and lumpectomy), as shown in the CONSORT flowchart (Fig. 1). One patient was excluded for meeting the exclusion criteria (i.e., the presence of chronic pain). A total of 49 patients were randomized to the study. The patients were allocated to the general anesthesia associated with SAM + PECS I protocol (n = 25) or the general anesthesia protocol (n = 24). All patients received the allocated intervention during the surgery, and the follow-up included evaluations in the PACU and at 24 h after surgery. Data from all patients were included in the analysis.

### Baseline characteristics

The randomized groups were compared, and the baseline characteristics of the patients are shown in Table 1. There were no differences between groups in age (U = 269.5, p = 0.54), weight (U = 298.0, p = 0.97) or body mass index – BMI (U = 246.0, p = 0.28). The women in the general anesthesia with the SAM block + PECS I group were statistically shorter than the women in the general anesthesia only group (U = 169.0, p = 0.04). There were no intraoperative or postoperative complications in the analyzed patient groups. The SF-36 eight scaled scores, including physical functioning, bodily pain, physical role functioning, general health perceptions, vitality, emotional role functioning, social role functioning and mental health, were evaluated before surgery. The Mann-Whitney test revealed no differences in scores between groups (U = 276.5, p = 0.64; U = 300.0; p = 1.00; U = 297.5; p = 0.96; U = 228.0, p = 0.15; U = 261.5, p = 0.44; U = 277.0, p = 0.64; U = 228.5, p = 0.15; and U = 262.5, p = 0.45). Considering comorbidities, there was no difference between groups considering patients taking different classes of medication (anxiolytics (Q_(1,24)_ = 0.14, p = 0.70), antidrepressive (Q_(1,24)_ = 1.80, p = 0.18), thyroids hormone (Q_(1,24)_ = 3.57, p = 0.59), medication to treat dyslipidemias (Q_(1,24)_ = 0.0, p = 1.00), proton pump inhibitors (Q_(1,24)_ = 0.0, p = 1.00), hormone replacement therapy (Q_(1,24)_ = 1.0, p = 0.37), antihypertensives (Q_(1,24)_ = 0.5, p = 0.48), estrogen receptor antagonists (Q_(1,24)_ = 1.0, p = 0.37), bronchodilators (Q_(1,24)_ = 0.0, p = 1.0), oral hypoglycemic drugs (Q_(1,24)_ = 0.2, p = 0.65), anticonvulsants (Q_(1,24)_ = 1.0, p = 0.32), muscle relaxants (Q_(1,24)_ = 1.0, p = 0.32) and corticosteroids (Q_(1,24)_ = 1.0, p = 0.32)).

**Table 1:** Demographic data of the population included in the clinical trial.

Parameters	General Anesthesia Patients’ Data				General Anesthesia & SAM block + PECS I Patients’ Data			
	Minimum	Maximum	Mean	Standard Deviation	Minimum	Maximum	Mean	Standard Deviation
Age (years)	44	74	54.83	10.49	31	72	56.2	11.33
Weight (kg)	49	82	66.87	7.49	49	103	66.86	12.22
Height (cm)	149	172	160.58	6.69	145	165	156.92	5.97
BMI	20.82	31.16	25.97	2.94	19.14	37.83	27.09	4.21
SF-36 Physical functioning	35	100	85.33	22.97	45	100	83.2	21.37
SF-36 Role limitations due to physical health	0	100	55.21	48.33	0	100	55	48.95
SF-36 Pain	22	100	81.88	26.47	0	100	80.9	28.35
SF-36 General health	20	80	50.21	15.07	35	90	57.6	15.88
SF-36 Energy/fatigue	20	100	66.88	23.02	25	100	62.2	19.58
SF-36 Social functioning	25	100	92.71	18.76	0	100	88	26.14
SF-36 Role limitations due to emotional problems	0	100	62.54	46.44	0	100	82.68	34.86
SF-36 Emotional well-being	20	96	67.83	19.07	24	100	64.48	20.24

Minimum, maximum, mean and standard deviation data collected from both groups of patients (general anesthesia and general anesthesia with SAM block + PECS I). The parameters measured were as follows: Age (years); Weight (kg); Height (cm); Body mass index (BMI); and Results of the SF-36 survey (divided into eight subtopics: Physical functioning; Role limitations due to physical health; Pain; General health; Energy/fatigue; Social functioning; Role limitations due to emotional problems; and Emotional well-being).

### Primary outcome - pain

**Figure 2:**
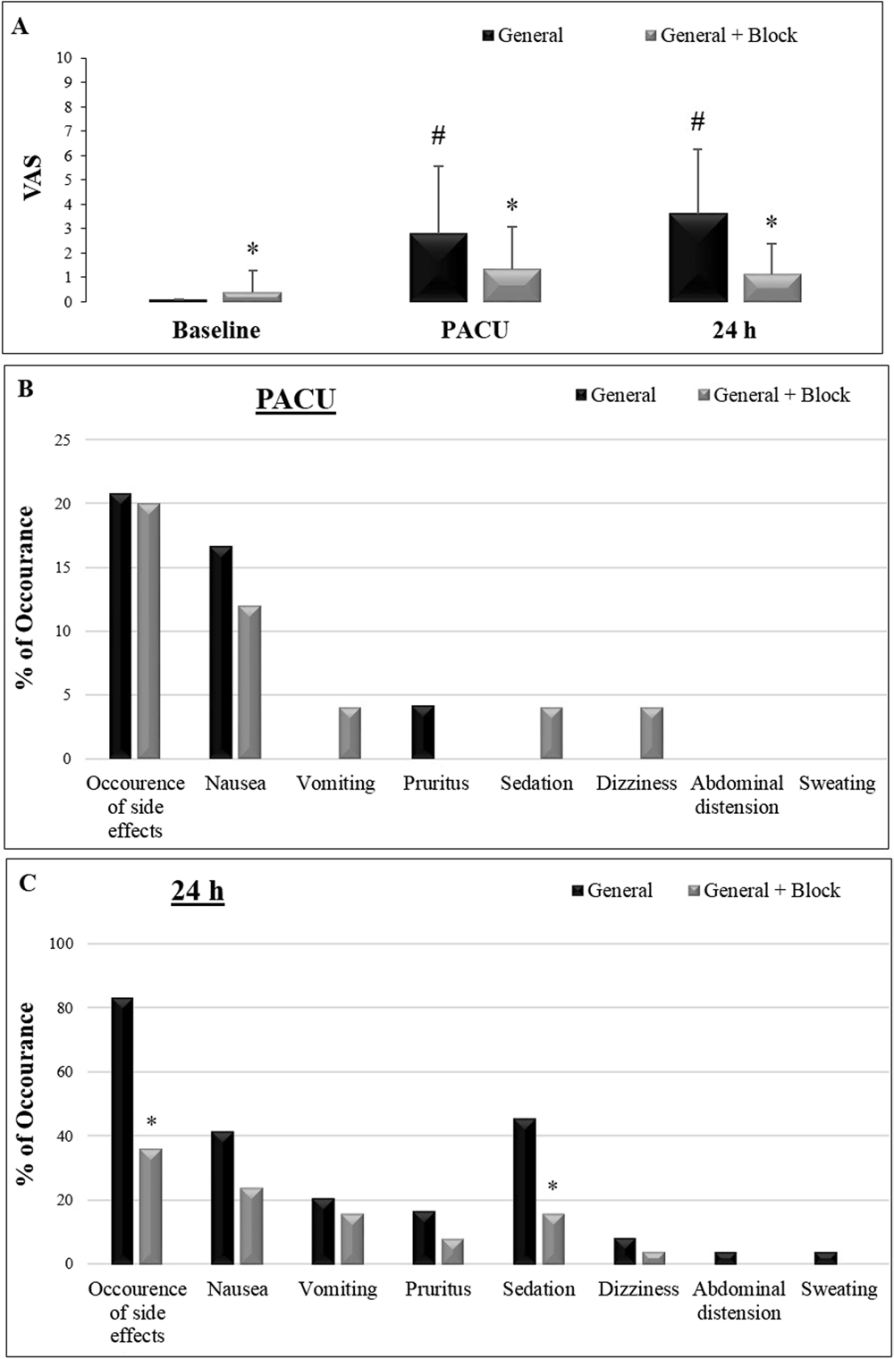
Pain and side effects measured in both groups: general anesthesia (N = 24) and general anesthesia with SAM block + PECS I (N = 25). (**A**) Pain levels measured using the Visual Analog Scale (VAS) at baseline, in the post-anesthetic care unit (PACU) and 24 h after surgery. (**B**) Percentages of side effects during the recovery period in the PACU. (**C**) Percentages of side effects 24 h after the anesthetic procedure. The data are presented as the mean ± standard deviation. *p < 0.05 compared to the general anesthesia only group. ^#^p < 0.05 compared to baseline.

Significantly lower VAS pain scores were recorded in the PACU and at 24 h after surgery in the general anesthesia associated with SAM + PECS I group compared to those in the general anesthesia only group. The general anesthesia only group exhibited increased pain scores in the PACU and at 24 h after surgery compared to baseline (Factor 1 group F_(2,140)_ = 212.8, p = 0,000001; Factor 2 Time F_(4,140)_ = 8.37, p = 0,000001; Interaction F_(4,280)_ = 4.07, p = 0.003; Fig. 2A).

### Secondary outcome - side effects

There were no block-related complications reported in the study. No indications of a block-related infection, such as purulent drainage, localized swelling, redness or heat, pain or tenderness at the site of injection, were reported.

In the PACU there were no differences in total side effects (Q_(1,24)_ = 0.0, p = 1.0), nausea (Q_(1,24)_ = 0.33, p = 0.56), vomiting (Q_(1,24)_ = 1.0, p = 0.32), pruritus (Q_(1,24)_ = 1.0, p = 0.32), sedation (Q_(1,24)_ = 1.0, p = 0.32), or dizziness (Q_(1,24)_ = 1.0, p = 0.32) as shown in Fig. 2B. There were no occurrences of abdominal distention or diaphoresis.

Total side effects (Q_(1,24)_ = 9.3, p = 0.002) and sedation (Q_(1,24)_ = 5.4, p = 0.02) decreased 24 h after surgery in the group that received SAM + PECS I association (Fig. 2C). There were no significant differences between groups in nausea (Q_(1,24)_ = 1.33, p = 0.25), vomiting (Q_(1,24)_ = 0.1, p = 0,74), pruritus (Q_(1,24)_ = 0.7, p = 0.41), dizziness (Q_(1,24)_ = 0.3, p = 0.56), abdominal distention (Q_(1,24)_ = 1.0, p = 0.32) or diaphoresis (Q_(1,24)_ = 1.0, p = 0.32).

### Secondary outcome - drug consumption

**Figure 3:**
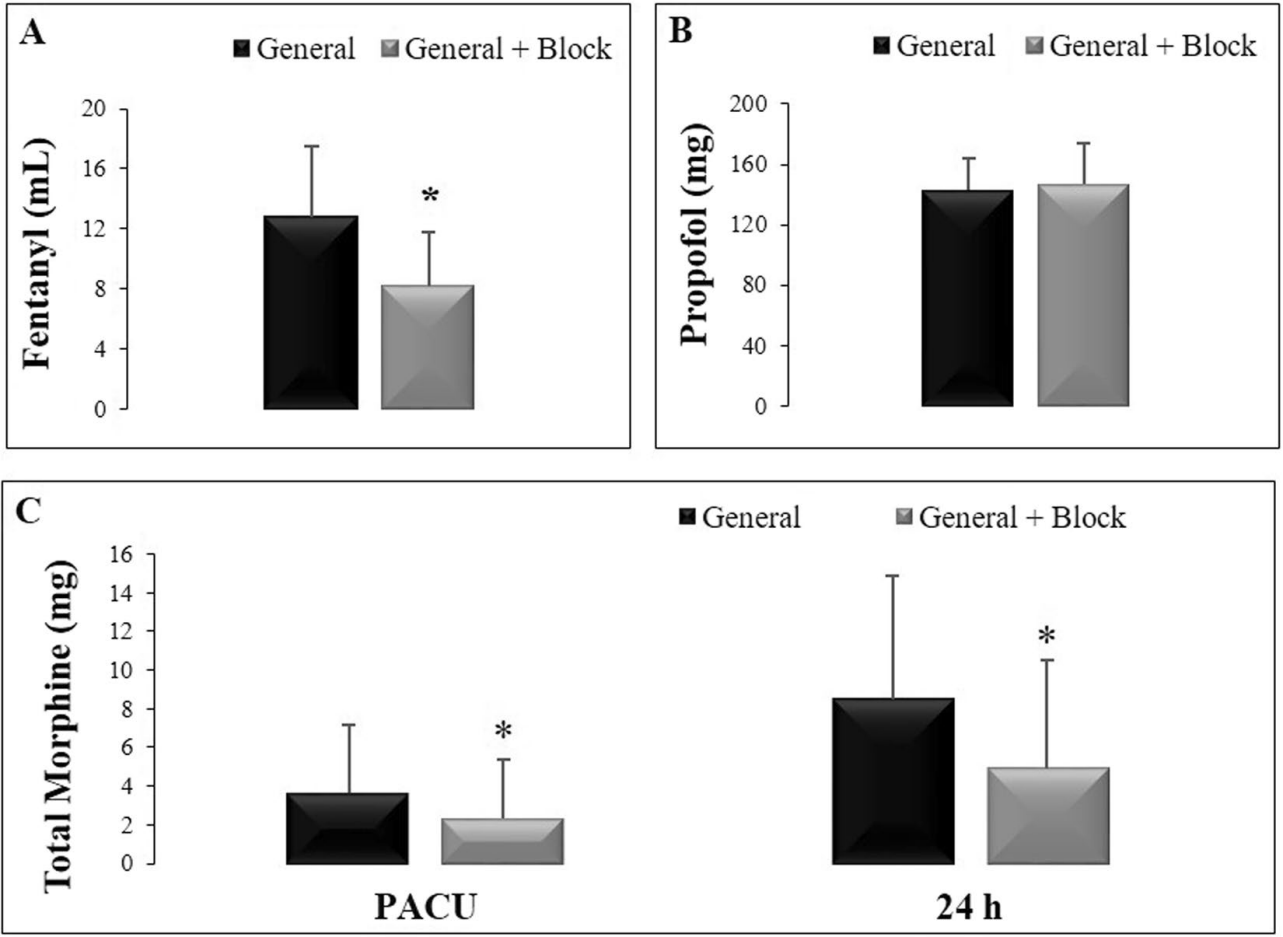
Amounts of drugs used during the surgical procedures in both groups: general anesthesia only (N = 24) and general anesthesia with SAM block + PECS I (N = 25). (**A**) Fentanyl consumption (mcg). (**B**) Propofol consumption (mg). (**C**) Amount of morphine (mg) consumed by the patients in both groups in the post-anesthetic care unit (PACU) and 24 hours after the anesthetic procedure. The data are presented as the mean ± standard deviation. *p < 0.05 compared to the general anesthesia only group.

There was a reduction in the intraoperative fentanyl requirement in patients who received general anesthesia associated with SAM + PECS I (U = 119.5, p = 0.0003) compared to patients who received general anesthesia only (Fig. 3A). The intraoperative requirements of propofol (Fig. 3B), cisatracurium and rocuronium were similar in both groups (U = 275.0, p = 0.62; U = 7.5, p = 0.20 and U = 147.0, p = 0.73).

Patients who received the SAM + PECS I block required less PCA-morphine in the PACU and at 24 h after surgery compared to patients who received general anesthesia only (Factor 1 group F_(2,93)_ = 139.5, p = 0,000001; Factor 2 Time F_(2,93)_ = 7.23, p = 0.000001; Interaction F_(2,93)_ = 0.68, p = 0.51), as shown in Fig. 3C.

### Secondary outcome - cytokines

**Figure 4:**
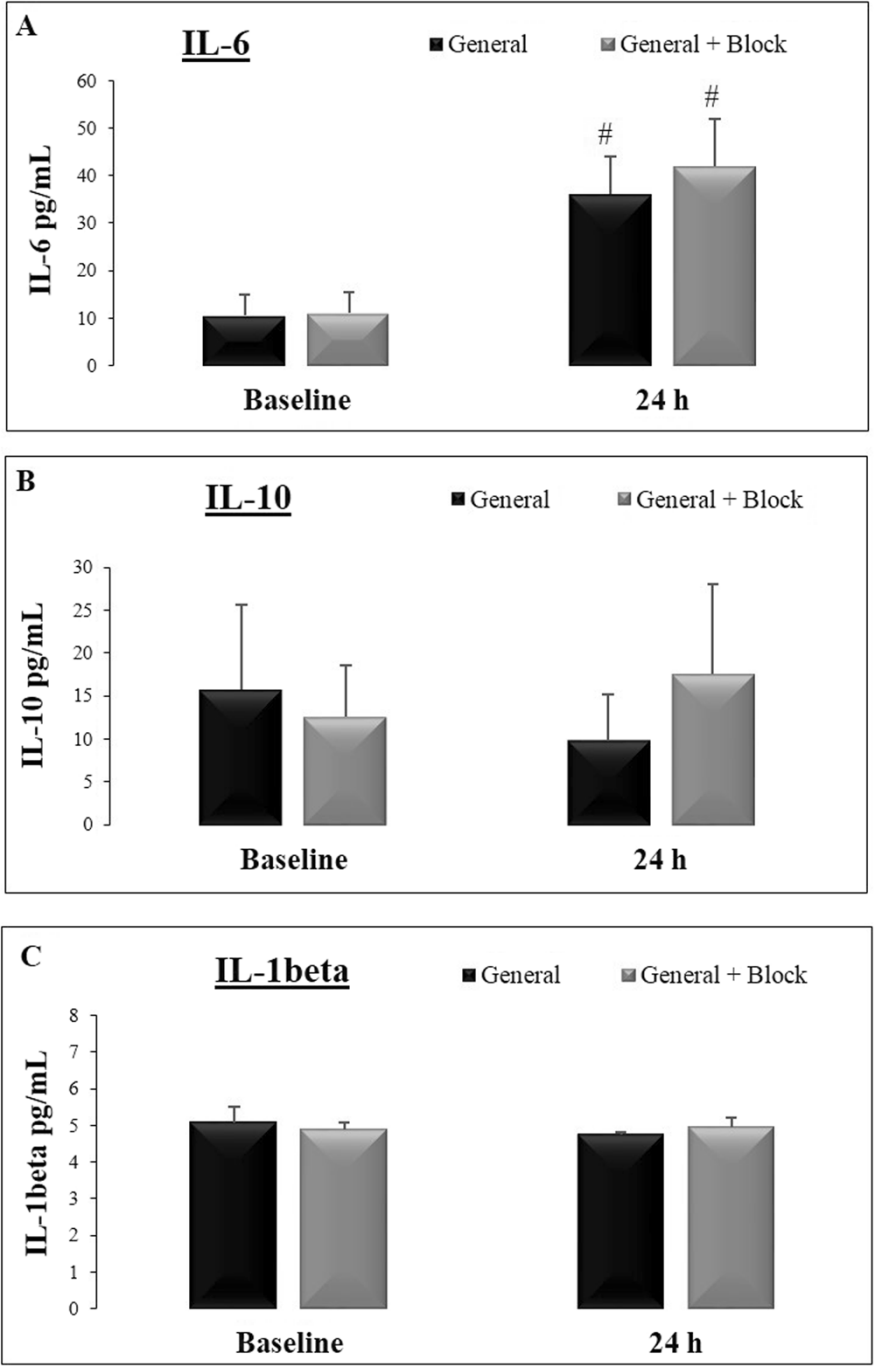
Quantification of serum cytokine levels (pg/mL) in the patients of both groups: general anesthesia only (N = 24) and general anesthesia with SAM block + PECS I (N = 25) at baseline and 24 h after the anesthetic procedure. (**A**) IL-6. (**B**) IL-10. (**C**) IL-1 beta. The data are presented as the means ± standard error. ^#^p < 0.05 compared to baseline.

Figure 4 shows the results for serum IL-6, IL-10 and IL-1 beta. Both groups exhibited an increase in IL-6 levels 24 h after surgery compared to baseline levels (Fig. 4A; Z = 3.9; p = 0.0001 and Z = 4.05; p = 0.0001 for general anesthesia and general anesthesia associated with SAM + PECS 1, respectively). There was no difference in IL-10 levels (Fig. 4B; Z = 1.32, p = 0.18 and Z = 1.34, p = 0.18, respectively) or IL-1 beta levels (Fig. 4C; Z = 1.00, p = 0.32 and Z = 0.45, p = 0.65, respectively).

### Additional data

There was no difference in PACU length of stay (U = 250,5; p = 0,32) or the duration of the anesthesia between groups (U = 258; p = 0,41). A Figure illustrating the ultrasound-guided SAM block and PECS I is available in the Supplementary Material (Supplementary Figure S1).

## Discussion

To our knowledge, this study is the first prospective, randomized study to report that general anesthesia associated with SAM + PECSI in radical mastectomy with axillary node dissection and breast reconstruction reduced the intraoperative consumption of fentanyl, the delivery of morphine in the PACU and at 24 h after surgery and decreased pain intensity and side effects.

The baseline characteristics of patients were not different in age, weight, BMI, or overall health status. However, there was a statistically significant difference in height between the groups. The risk factors for breast cancer include weight and BMI^22,23^. A difference in height is not a parameter that would affect the results of regional anesthesia because the most important aspect for calculating the amount of anesthesia is the weight^24,25^.

No block-related complications occurred during the study. In the literature, the incidence of complications is very low, and ultrasound was reportedly a fundamental tool that provided significant improvements in regional anesthesia in breast surgeries, encouraging its iincorporation into the clinical practice for interfascial chest wall blocks^26–29^.

The association of SAM + PECS I with general anesthesia reduced morphine consumption in the PACU and at 24 h after the procedure. Previous studies have demonstrated that PECS or SAM block reduced opioid consumption in breast cancer surgery^15,17,30,31^. Intraoperative high doses of opioids are associated with long-term chronic pain after mastectomy^32^.

The literature regarding regional anesthesia reports no differences in propofol, cisatracurium and rocuronium consumption^33–35^, which supports our data.

The use of SAM block + PECS I decreased pain scores in the PACU and at 24 h after the procedure. An effective postoperative pain control is an essential component to avoid chronic pain, which exhibits very high rates in breast cancer patients after surgery^3^. The role of regional anesthesia in the management of postoperative pain is well known^36^. Specifically, one case report evaluated PECS block for various types of breast surgeries, and the result revealed an excellent intraoperative and postoperative analgesia^37^. A cadaver study suggested that PECS I block produced greater analgesia of the axillary region compared to PECS II block^38^.

Our data demonstrated that the association SAM + PECS I reduced the occurrence of side effects. Consistent with our data, Bashandy and Abbas reported that the use of PECS in mastectomy decreased nausea, vomiting and sedation compared to the use of general anesthesia^17^. A recent meta-analysis supported that regional anesthesia decreased anesthesia side effects^30^.

Cytokines exert inhibitory or excitatory effects on tumor growth depending on their concentrations in the tumor microenvironment, and systemic levels of these mediators may correlate with the disease stage and progression^39^. Furthermore, patients with breast cancer have impaired immune systems^40^. However, no studies have evaluated inflammatory mediators after the association of SAM block + PECS I during radical mastectomy. Our results revealed higher serum levels of IL-6 24 h after surgery, suggesting that the surgical procedure itself was responsible for the increase. This hypothesis is supported by the fact that the surgical procedure modulates the immune system, especially in patients with malignant diseases^41^. Therefore, mastectomy increases IL-6 in cancer-associated adipocytes^42,43^.

There were no differences between groups (general anesthesia versus general anesthesia associated with SAM + PECS I) in IL-6, IL-10 and IL-1 beta levels. Mettler *et al*. evaluated blood samples of mastectomy or segmental resection patients and demonstrated no difference in the level of IL-1 beta as a consequence of the surgical procedure^41^. Additionally, data from the literature comparing the effects of general anesthesia and regional spinal/epidural anesthesia in different types of surgeries revealed no difference in IL-1 beta levels compared to anesthesia or the levels before and after the procedure^44,45^. No difference in the anti-inflammatory IL-10 level was observed between groups or before and after the surgical procedure. Tumor cells secrete IL-10^46^ especially in breast cancer^47,48^ and in the context of the surgery T lymphocytes are the main source of IL-10 release^49^, which may explain the consistent IL-10 levels in our results. One study using paravertebral regional anesthesia reported decreased in IL-1 beta levels and a modest increase in IL-10 levels (10%) without affecting IL-6 compared to the non-blocking group^5^. One of the main reasons for this discrepancy may be the study design, which compared propofol/paravertebral to sevoflurane/opioid. The type of hypnotic used to maintain anesthesia may also affect the cytokine profile. Specifically, IL-1 beta levels increase when sevoflurane is used compared to propofol^50^. Our study reported that serum cytokines exerted a minor influence on the possible beneficial effect of the regional anesthesia on clinical outcomes, as previously suggested^44^.

Surgery is the first choice of treatment, and one of the goals of performing regional anesthesia may be the reduction of post-mastectomy pain syndrome^51^. Regional anesthesia may be an important tool in the reduction of tumor growth and metastasis recurrence^52^. It has been implicated that damage to intercostobrachial nerve could be responsible for persistant pain after breast cancer surgery and the local anesthetic block of this region could be an alternative to prevent this type of pain^53^. Supporting this view, Takimoto demonstrated that SAM block was a treatment modality for chronic neuropathic pain after partial mastectomy^54^.

One limitation of our trial includes the impossibility of measuring the hospital length of stay after surgery. Since no complications were noted, all mastectomy patients were discharged from the hospital three days after the procedure as part of the standardized protocol of the hospital. In addition, serum levels of ropivacaine were not evaluated, which may represent a bias because there are no studies indicating a possible anti-inflammatory effect of ropivacaine.

In conclusion, our results suggest that SAM + PECS I block in association with general anesthetic provides the most effective analgesia for radical mastectomy, but further studies are required.

### Implications

This report is the first prospective, randomized, controlled study to report the benefits of general anesthesia associated with SAM block + PECS I in radical mastectomy with breast reconstruction. Our results highlight the importance of improving anesthetic protocols for radical mastectomy and further examining of the benefits of regional anesthesia and pain reduction on the chronification process.

## Methods

### Patients

The study was a single-center, prospective, randomized controlled clinical trial. The patients participating in the trial were randomly assigned to the group receiving general anesthesia only (standard treatment) or the group general anesthesia associated with SAM block + PECS I. The block randomization method was designed to randomize subjects into groups. It was generated by the corresponding author and included sealed envelopes, and the patients were blinded to this process. An anesthesiologist recruited participants from our ambulatory care unit. The informed consent was provided to all prospective study subjects. The Ethics in Research Committee of the Hospital Sirio Libanes/Brazil Platform approved the project (CAAE 48721715.0.0000.5461), it is registered at Registro Brasileiro de Ensaios Clinicos (ReBEC), ClinicalTrials.gov, Identifier: NCT02647385, and the trial is registered under the name: ‘Pain Control in Breast Surgery: Analgesia, Opioid Consumption and Inflammatory Response Evaluation’. The principal investigator’s name is Raquel Chacon Ruiz Martinez and the date of registration is 12/28/2015. All methods were performed in accordance with the relevant guidelines and regulations.

The inclusion criteria were female patients aged 18–75 years old who provided written consent, with American Society of Anesthesiology (ASA) physical status I or II, and who were suitable for elective radical mastectomy with axillary node dissection and breast reconstruction. Exclusion criteria were previous allergy to medications used in the study, history of mental disorders and chronic pain. Chronic pain was diagnosed using the Douleur Neuropathique 4 (DN4) questionnaire^55^. All data were entered into the REDCap (Research Electronic Data Capture) database.

### Surgery and postoperative analgesia

Three different surgical teams performed or supervised all surgery. All patients received a standardized postoperative analgesic regimen that consisted of regular metamizole (1 g every 6 h), ketoprofen (100 mg every 12 h) and patient-controlled analgesia (PCA) rescue with intravenous morphine at the end of the surgical procedure using a program of a 2 mg bolus, an interval of 10 min and a maximum dose of 12 mg per hour. The total consumption of opioid was recorded.

### Clinical assessments

Demographic data and quality of life parameters were acquired from medical records and an interview with each subject at the time of study enrollment. The short-form health survey (SF-36) was assessed as an indicator of overall health status.

### Primary outcome

The primary outcome measure was pain intensity, which was assessed using visual analog pain scale (VAS) scores before surgery, in the post-anesthetic care unit (PACU) and at 24 h after surgery. The VAS scores range from 0–10, and a blinded experimenter evaluated the VAS scores.

### Secondary outcomes

Blinded observers evaluated secondary outcomes, which included the consumption of fentanyl and propofol in the intraoperative room, the consumption of PCA-morphine in the PACU and at 24 h after the surgery, the time spent in the PACU, the side effects exhibited in the PACU and at 24 h after the surgery, and serum cytokines IL-1 beta, IL-6 and IL-10 levels before and 24 h after surgery. Any complications were recorded.

### General anesthesia

The patients received 7.5 mg of midazolam orally as premedication one h before surgery. Anesthesia was induced with fentanyl (2–3 mcg kg^−1^), propofol (2–3 mg kg^−1^) and cisatracurium (0.1 mg kg^−1^) or rocuronium (0.6 mg kg^−1^). Anesthesia was maintained with sevoflurane (1.5–2.0%) and 50% oxygen and air with positive pressure ventilation in a circle system. Additional fentanyl boluses were administered if necessary.

### SAM Block + PECS I

Three anesthetists who were experienced in performing SAM and PECS I performed these blocks. Prior to the procedure, 2% chlorhexidine solution with 70% isopropyl alcohol and sterile ultrasound conducting gel were applied to the skin. A nonsterile conductive gel was applied to the face of the transducer. The operator wore sterile gloves and applied a sterile cover to the transducer. Before and after each procedure, the ultrasound apparatus was appropriately cleaned and disinfected with disinfectant wipes and allowed to air dry. A 12.5-MHz linear probe was positioned in the midaxillary line at the level of T5, and an in-plane needle was inserted into the fascia between the latissimus dorsi and serratus anterior muscles for ropivacaine (20 mL of 0.375%) injection. The injection for PECS I was performed in the fascia between minor and major pectoral muscles using 10 mL of 0.375% ropivacaine^8,11^. Needle position was confirmed using visualization of the separation of the layers with dispersion of the injected volume.

### ELISA

Blood samples were collected in EDTA-Vacutainer tubes and processed by the Biobanco unit from the Hospital Sirio Libanes. Blood was immediately placed on ice and centrifuged. The plasma was separated and stored at −80 °C until measurement. IL-1beta, IL-6 and IL-10 concentrations in samples were measured in duplicate using specific commercial enzyme-linked immunosorbent assay (ELISA) kits (R&D Systems). The ELISA protocol was performed according to the manufacturer’s specifications.

### Study design

Consenting patients were interviewed on the day of surgery, and the SF-36, DN4 and VAS scales were used. A blood sample was collected before to surgery. Anesthesia was performed according to group randomization (general anesthesia only or general anesthesia associated with SAM + PECS I), and the consumptions of fentanyl, propofol, cisatracurium and rocuronium were recorded. Patients were admitted to the post-anesthetic care unit (PACU) after surgery, and the following parameters were measured: time spent in PACU, VAS, PCA-morphine consumption, side effects and complications. Blood samples were collected 24 h after surgery, and VAS, PCA-morphine consumption, side effects and complications were assessed. Blood samples were evaluated in the ELISA assay to determinate the cytokine levels.

### Duration of the study

The surgeries were performed from December 2015 to June 2016.

### Safety Considerations and Follow-up

Patient safety was monitored during the study. All adverse events were monitored throughout the study.

### Breaking the codes

At the end of the study, the sponsor of the study had permission to release the code break envelopes/randomization list. These information codes were broken once the trial database was completed.

### Data availability

The datasets generated and/or analyzed during the current study are available from the corresponding author upon reasonable request in the REDCap database (https://redcap.iephsl.org.br).

### Sample size justification and statistical analysis

The sample size was calculated using www.openepi.com based on the results of Wu, who evaluated general anesthesia plus paravertebral versus general anesthesia block and produced an effect size of 1.5 for VAS^46^. Sixteen patients per group were required to achieve significant results with an alpha of 0.05 and a beta of 90%. There are no previous data on general anesthesia versus SAM + PECS I. Therefore, we estimated that 50 patients divided into two arms would be sufficient to show an effect of regional anesthesia versus general anesthesia.

Data are shown as the means ± standard deviation and Elisa data are shown in mean ± standard error. Demographic data, quality of life, drug consumption and time spent in the PACU were analyzed using the Mann-Whitney test. VAS and opioid consumption were analyzed using two-way ANOVA repeated measures (Factor 1: group, Factor 2: time) followed by Newman-Keuls post-hoc tests. The interleukin data were analyzed using the Wilcoxon test. Medication and side effects were analyzed using Cochran’s Q test. Significance was set at P ≤ 0.05.

## Electronic supplementary material

Supplementary Figure
