# Supplementary material for: Benefits in radical mastectomy protocol: a randomized trial evaluating the use of regional anesthesia
Source: Sci Rep. 2018 May 18;8:7815. doi: 10.1038/s41598-018-26273-z (PMC5959858; doi:10.1038/s41598-018-26273-z)
Supplement: Supplementary file 1 — Supplementary Figure [file 41598_2018_26273_MOESM1_ESM.docx]

**Supplementary info**

**Benefits in radical mastectomy protocol: a randomized trial evaluating the use of regional anesthesia**

Marcio Matsumoto^1,2^, Eva M. Flores^1,2^, Pedro P. Kimachi^1,2^, Flavia V. Gouveia^1^, Mayra A. Kuroki^1^, Alfredo C.S.D. Barros^1^, Marcelo M.C. Sampaio^1^, Felipe E.M. Andrade^1^, João Valverde^1,2^, Eduardo F. Abrantes^1^, Claudia M. Simões^1,2&^, Rosana L. Pagano^1&^, Raquel C.R. Martinez^1&*^

**Supplementary Fig. S1**


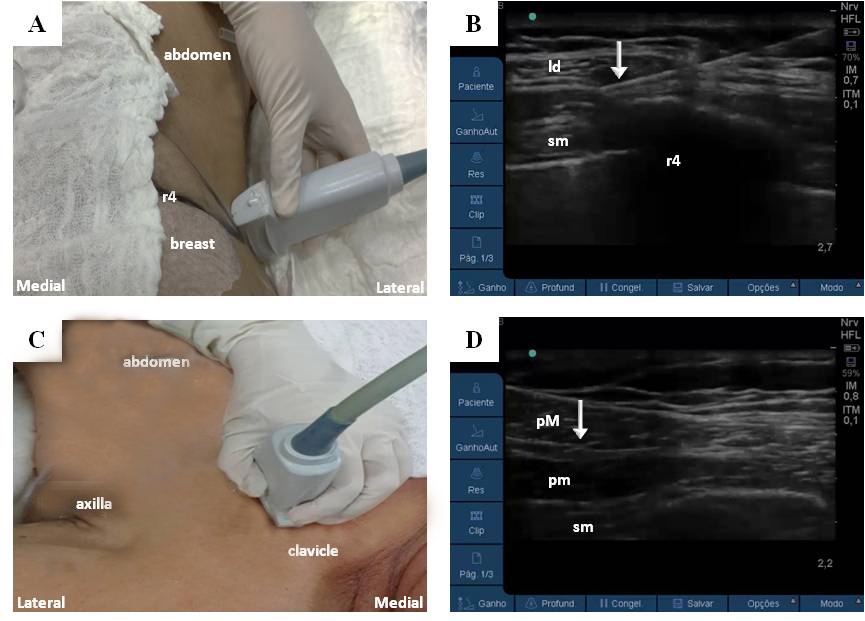


**Supplementary Fig. S1.** Procedure for general anesthesia with SAM block+PECS I. **a.** Illustrative image of the position of the ultrasound probe for the SAM block. **b.** Ultrasound image of the position of the needle into the fascia between the latissimus dorsi (LD) and serratus anterior muscles (SM). **c.** Illustrative image of the position of ultrasound guidance for PECS I. **d.** Ultrasound image of the position of the needle in the fascia between the minor (Pm) and major pectoral muscles (PM). The white arrow indicates the location of the needle. Lateral: orientation lateral; Medial: orientation medial; r4: fourth rib.
